# Occurrence of Ergot Alkaloids in Major and Minor Cereals from Northern Italy: A Three Harvesting Years Scenario

**DOI:** 10.1021/acs.jafc.3c05612

**Published:** 2023-10-16

**Authors:** Laura Carbonell-Rozas, Arianna Alabrese, Raffaele Meloni, Laura Righetti, Massimo Blandino, Chiara Dall’Asta

**Affiliations:** † Department of Food and Drug, University of Parma, Viale delle Scienze 27/A, Parma 43124, Italy; ‡ Department of Agricultural, Forest and Food Sciences, University of Turin, Largo Paolo Braccini 2, Grugliasco 10095, Italy; § Laboratory of Organic Chemistry, Wageningen University, Wageningen 6708, The Netherlands; ∥ Wageningen Food Safety Research, Wageningen University & Research, Wageningen 6700, The Netherlands

**Keywords:** ergot alkaloids, cereals, occurrence study, experimental crops

## Abstract

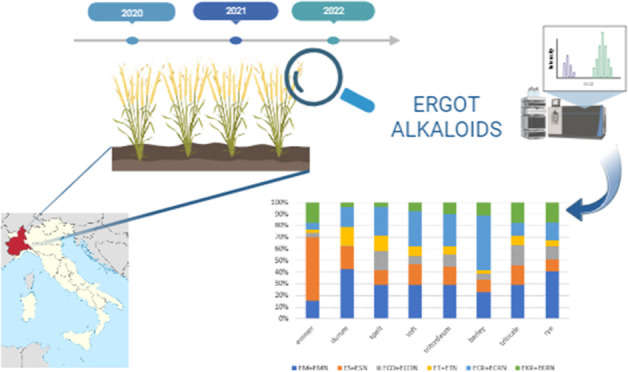

Ergot alkaloids (EAs), mycotoxins produced mainly by fungi of the *Claviceps* genus, have been frequently reported in rye, while their increasingly frequent occurrence in other cereals is likely related to weather conditions, with the incidence of ergot sclerotia in winter grains being related to heavy rainfall and moist soils at critical periods. However, compared to other regulated mycotoxins, data about the prevalence and occurrence of EAs in major and minor cereals harvested in the Mediterranean growing areas are still scant. In this regard, the current study reported the occurrence of EAs in 18 genotypes of winter cereals harvested over 3 years from an experimental field located in North Italy which were analyzed by HPLC–MS/MS. Results indicate a widespread occurrence of all the major EAs in all the considered cereal crops, especially under supportive meteorological conditions. EA contamination was dependent on the harvest year (*p* < 0.0001) which was particularly high in 2020 for all the considered species. The results also demonstrated a large co-occurrence of EAs with 98 cereal samples out of 162 contaminated with at least one of the 12 EAs (60% positive samples) in the range LOD: 15,389 μg/kg (median value: 2.32 μg/kg), expressed as the sum of the EAs. Rye was confirmed to be the crop more susceptible to the fungal infection (EAs content up to 4,302 μg/kg). To the best of our knowledge, we have reported the accumulation of EAs in tritordeum (LOD: 15,389 μg/kg) and in emmer (LOD: 1.9 μg/kg) for the first time.

## 1 Introduction

Ergot alkaloids (EAs) are mycotoxins produced mainly by fungi of the *Claviceps* genus, most notably by *Claviceps purpurea*, which can parasitize susceptible host plants such as grasses, rye, triticale, wheat, oat, and barley.^1,2^ The growing grain or seed is replaced with fungal structures known as sclerotia that contain EAs whose content shows significant variations depending on several factors such as the maturity of the sclerotia, the fungal strain, the host plant, level of epimerization, the geographical region, and weather conditions.^3−5^

EAs can be found in cereals and milling products following the sclerotia breaking at harvest and postharvest stage.^2^ Their biological activity is well documented over time and may lead to relevant adverse effects in animals and humans following both acute and chronic exposure.^6^ Although improvements in agricultural techniques have considerably reduced the presence of EAs in cereals, their occurrence in cereals and products thereof is frequently reported, especially in winter grains.

Due to analytical limitations, ergot contamination in grains has been monitored for a long time by determining the presence of ergot bodies in cereals. However, this approach does not provide reliable information for risk assessment as sclerotia may significantly vary in size, weight, and composition. Thus, the development of proper EA-targeting analytical protocols was encouraged instead.^6^

EA-producing fungi are characterized by a large chemodiversity,^7^ with more than 80 different ergot alkaloids identified in grains infected with *Claviceps* spp. Common EA structures are divided into ergopeptine and ergoline alkaloid subfamilies. In addition, alkaloids containing a C9 = C10 double bond easily epimerize with respect to the center of symmetry C8 depending on temperature and pH.^1^ Epimerization may also occur during heat processing, such as pelleting in feed production.^8^ Therefore, the European Food Safety Authority (EFSA) has recommended to focus the monitoring on the six main epimer pairs produced by *Claviceps* spp., namely, ergometrine (EM), ergometrinine (EMN), ergosine (ES), ergosinine (ESN), ergotamine (ET), ergotaminine (ETN), ergocornine (ECO), ergocorninine (ECON), a mixture of α- and β-isomers of ergocryptine (EKR) and ergocryptinine (EKRN), ergocristine (ECR), and ergocristinine (ECRN) in relevant food and feed commodities. The -inine epimers are described to be biologically inactive; however, due to the frequent interconversion under common conditions, both forms (-ine and -inine) have been included in the EFSA risk assessment.^6,9^ Thus, the stability and degree of epimerization of the six major EAs have to be considered during their analysis.^10^ In this regard, a great variety of analytical methods to determine the main EAs together with their corresponding epimers have been developed and summarized in several reviews.^11−13^

In 2023, the European Commission published Regulation (EU) 915/2023,^14^ setting the maximum permitted limits for the sum of the above-mentioned 12 EAs in a range of cereals and food thereof. Maximum permitted values are in the range of 100–500 μg/kg for milling products obtained from rye, barley, wheat, spelt, and oats (which will be further decreased to 50–250 μg/kg from January 2024) and 20 μg/kg for processed cereal-based food for infants and young children.

In spite of the analytical challenges, the recent regulatory framework has prompted a number of studies focused on the EA occurrence in the regulated cereals harvested and processed in Europe.^15−21^ While EAs have been frequently reported in rye (*Secale cereale*) over time, their increasingly frequent occurrence in other cereals is likely related to climate change scenarios,^22,23^ being the incidence of ergot sclerotia in winter grains related to heavy rainfall and moist soils at critical periods.^1,22^ Rye, an open pollinator plant, is considered the most susceptible grain to ergot, followed by self-pollinators such as wheat (*Triticum* spp.), triticale (×*Triticosecale*), barley (*Hordeum vulgare*), and oats (*Avena sativa*).^24^ Fungal growth in rye has an optimum at 18–20 °C, although it has been described already at 9–15 °C, and sclerotium formation is favored under cool, wet weather conditions, especially during the flowering stage.^2^

Based on the body of evidence, EA levels in food products are rather low due to the efficient mitigation strategies applied at milling plants. On the other hand, the high presence of sclerotia in unprocessed grains may affect animal exposure through contaminated feed, especially following the upcycling of milling byproducts.^8,25,26^ However, compared to other regulated mycotoxins, data about the prevalence and occurrence of EAs in major and minor cereals harvested in the Mediterranean growing areas are still scant and varieties of commercial interest are poorly explored for their potential resistance/susceptibility.

In this regard, the current study aims to assess the occurrence of EAs in winter cereals collected over three harvest years from experimental fields located in Northern Italy. Overall, 18 genotypes belonging to 8 major and minor cereal species were considered; among them 3 varieties of tritordeum (×*Tritordeum martini*), a new amphidiploid hybrid species derived from the cross between durum wheat (*Triticum turgidum* spp. durum), and a wild barley (*H. chilense*) were studied.^27^ To the best of our knowledge, the potential accumulation of EAs in tritordeum and emmer (*T. turgidum* spp. dicoccum) has never been considered before.

## 2 Materials and Methods

### 2.1 Chemical and Reagents

All reagents were of analytical reagent grade, and solvents were of LC–MS grade. Acetonitrile (MeCN), methanol (MeOH), ammonium carbonate, and formic acid were supplied by VWR International (Milan, Italy). Z-sep+ sorbent for cleanup was obtained from Supelco (Bellefonte, PA, USA), while the C18 sorbent was supplied by Agilent Technologies (USA).

### 2.2 Standards

Standards of ES, ECO, EKR, ECR, and the corresponding epimers, ESN, ECON, EKRN, and ECRN, were purchased from Techno Spec (Barcelona, Spain), whereas EM, ET, EMN, and ETN were obtained from Romer Laboratories (Getzersdorf, Austria). Following the indications of the manufacturer, the standards were reconstituted in 5 mL of MeCN to achieve concentrations of 500 μg/mL for the main EAs and 125 μg/mL for the epimers. Immediately after that, intermediate dried stock solutions were prepared taking aliquots of the individual or mixed standard solutions and drying them under a gentle stream of N_2_. Afterward, the intermediate dried stock solutions were stored at −20 °C and reconstituted in the required amount of MeCN just its use.

### 2.3 Samples

Cereal species were grown side by side over three growing seasons (harvest years 2020, 2021, and 2022) on the same experimental field located in Cigliano, Italy (Piedmont; 45° 18′ N, 8° 01′ E; altitude 237 m), in shallow and sandy-loam soil (Italy). A total of 18 genotypes belonging to 8 crops were considered including diploid, tetraploid, and hexaploidy species, as reported in Table S1. The genotypes were assigned to experimental units using a completely randomized block design with a 10.5 m^2^ plot (7 × 1.5 m) and three replications. The daily temperatures and precipitations were measured at a meteorological station located near the experimental area (Table S2).

The same agronomic technique was adopted for all genotypes according to the common management of winter cereal in the growing areas. Briefly, the previous crop in all of the year was maize, and the field was plowed (25 cm) in autumn, incorporating the debris into the soil, and this was followed by disk harrowing to prepare a suitable seedbed. Sowing was conducted in 12 cm wide rows at a seeding rate of 200 (hybrid rye, cultivar Su Nasri and Su Performer), 300 (conventional rye, emmer, and spelt), and 450 (soft and durum wheat, barley, triticale, and tritordeum) seeds m^–2^ at the beginning of November. All plots received 80 kg ha^–1^ of nitrogen, applied as ammonium nitrate, and split equally at tillering and stem elongation. The weeds were chemically controlled a mesosulfuron-methyl and iodosulfuron-methyl-sodium at stem elongation. No fungicide treatment was carried out to control Fusarium head blight infection at flowering. Harvesting was carried out in the first decade of July using a Walter Wintersteiger cereal plot combine harvester.

Representative subsamples (2 kg) were collected from each plot at harvest. The number of ergot sclerotia was visually counted in 500 g grain samples and expressed as g of sclerotia per kilogram of grain, according to the Commission Regulation (EU) 2023/915. All grain samples, without any dehusking operation for emmer, spelt, and barley, were ground to whole-meal using a laboratory centrifugal mill equipped with a 1 mm sieve (Model ZM-200, Retsch, Haan, Germany). Collected samples were analyzed for EAs considering 3 biological replicates each, for a total of 162 samples over three years.

### 2.4 Instrumentation and Equipment

HPLC–MS/MS experiments were performed in a Dionex Ultimate 3000 Autosampler HPLC coupled to a triple quadrupole mass spectrometer (TSQ Vantage; Thermo Fisher Scientific Inc., San Jose, CA, USA Triple) equipped with an electrospray ion source (ESI). Chromeleon HPLC and X-Calibur software were used for acquisition and data analysis, respectively. During the sample treatment procedure, a vortex HS 501 digital IKA-WERKE (Staufen im Breisgau, Germany) and an Eppendorf 5810 R centrifuge (Hamburg, Germany) were also used.

### 2.5 Sample Preparation for the Extraction of Ergot Alkaloids

A previously optimized sample treatment for the extraction of EAs from oat-based functional foods was employed.^28^ Briefly, a portion of 1 g of the homogenized sample of each cereal type was placed into a 15 mL falcon tube with a conical bottom, and then, the extraction solution composed of 4 mL of MeCN and 5 mM ammonium carbonate (85:15, v/v) were added. Basic conditions were needed in order to avoid rapid epimerization of the compounds. Then, the mixture was horizontally shaken for 10 min, and afterward, the sample was centrifuged at 9000 rpm for 10 min at 4 °C. Subsequently, the whole upper layer was collected and placed into a 15 mL falcon tube containing 150 mg of a mixture of C18:Z-Sep+ (1:1) as dispersive sorbent for the cleanup step. Then, the 15 mL tube was vigorously shaken for 10 min and centrifuged at 9000 rpm for 10 min at 4 °C. Finally, the upper layer was fully transferred to a glass tube and evaporated to dryness under a gentle stream of N_2_. The residue was reconstituted with 750 μL of a mixture of MeOH/water (50:50, v/v), and 0.2 μL as the injection volume was injected into the HPLC–MS/MS system.

### 2.6 HPLC–MS/MS Conditions

The chromatographic separation of EAs was carried out using a C18 Kinetex column (100 mm × 2.1 mm, 2.6 μm). The mobile phase consisted of 0.3% formic acid aqueous solution (solvent A) and MeOH with 0.3% formic acid (solvent B) at a different flow rate. The eluent gradient profile was as follows: 0–2 min 30–70% B at a flow rate of 0.3 mL/min; 2.1–9 min 30–70% at 0.5 mL/min B; 9–11 min 10–90% B at 0.5 mL/min B; 11–11.5 min 30–70% B at 0.3 mL/min. The column temperature was set at 40 °C and the injection volume was 2 μL. To minimize epimerization, the injection sample sequence was limited to 12 h. Moreover, control standard solutions of EAs were injected at the beginning, middle, and end of each analysis sequence.

The mass spectrometer was operated in the positive electrospray ionization (ESI+) mode under the selective reaction monitoring (SMR) conditions, which are shown in Table S3. The monitored ions as precursor ions were the protonated molecules [M + H]^+^ in all cases. In addition, two product ions were studied for each mycotoxin.

The spray voltage was 3000 V, the capillary temperature was set at 270 °C, the vaporizer temperature was set at 300 °C, the sheath gas flow was set at 50 units, and the auxiliary gas flow was set at 15 units. The collision energies were optimized during the infusion of analyte standard solutions (1 mg/kg, in MeOH) by employing an automatic function of X-Calibur software (Thermo Fisher Scientific).

### 2.7 Statistical Analysis

Statistical analyses were performed using an XLSTAT2022 (Lumivero, Denver, CO, USA). Data were log-normalized before analysis and analyzed by Full Factorial ANOVA.

## 3 Results and Discussion

### 3.1 Yearly Occurrence of Total EAs

A total of 162 grain samples were collected over three harvest seasons (2020, 2021, and 2022) from experimental fields located in the North of Italy. The sample set was analyzed for the regulated Eas and potential effects due to the climate (factor 1: harvest season) and to the genotype (factor 2: species). Furthermore, within the most contaminated species, the differences in EA accumulation among the varieties have been explored.

Regarding the incidence, 98 cereal samples out of 162 were found to be contaminated with at least one of the 12 EAs (60% positive samples). Table 1 summarizes the incidence of contamination over the three harvesting years together with the amount of sclerotia found in each cereal type.

**Table 1:** Incidence of Ergot Sclerotia and Total EAs Contamination in Grain of Different Cereals under Three Growing Seasons^a^

	2020		2021		2022	
crop	Ergot sclerotia (g/kg)	incidence of sample with EAs > LOQ (%)	Ergot sclerotia (g kg^–1^)	incidence of sample with EAs > LOQ (%)	Ergot sclerotia (g/kg)	incidence of sample with EAs > LOQ (%)
emmer	0	0	0	67	0	0
durum wheat	0	75	0	0	0	0
spelt	0	100	0	0	0	0
soft wheat	0	100	0	78	0	0
tritordeum	0	100	1.32	100	0.03	22
barley	0	50	0	17	0.03	17
triticale	0	100	0	67	0	0
rye	1.97	100	0.14	100	0.18	89

a (*a* = samples containing one or more individual EAs at concentrations equal to or above their corresponding LOQ were considered positives; *b* = incidence rate of contamination).

Interestingly, although sclerotia have never been observed in *Triticum* species (i.e., emmer, durum, and soft wheat), EAs are present in such samples also reaching concentrations in soft wheat above the MLs which will be enforced in 2024. Notably, sclerotia content above the legal limits (0.2 g/kg) was observed only in 2020 in rye and in 2021 in tritordeum, when extremely high EAs contents were found (i.e., up to 4302 μg/kg and to 15,389 μg/kg in rye and tritordeum, respectively). Consistently with the previous literature, a correlation between sclerotia and Eas concentration cannot be easily drawn, and this underlines one more time the need for analytical determination of EAs instead of sclerotia counting as a ground for compliance verification.

EAs contamination in cereal samples across the 2020–2021–2022 seasons was found in the range LOD: 15,389 μg/kg (median value: 2.32 μg/kg), expressed as the sum of the EAs at the lower bound. Based on a full factorial ANOVA, EAs contamination was dependent on the harvest year (*p* < 0.0001) and on the species (*p* < 0.0001), as well as for the interaction between factors (*p* < 0.0023). Aggregated results based on species are reported in Table 2, while the full data matrix including varieties is available as Supporting Information (Table S4).

**Table 2:** Total EA Concentrations, Expressed as the Sum of the 12 Monitored EAs and Found in the Considered Cereals over the Harvesting Years (LOD ≤ 0.05 μg/kg)

	2020		2021		2022	
crop	range (μg/kg)	median (μg/kg)	range (μg/kg)	median (μg/kg)	range (μg/kg)	median (μg/kg)
emmer	LOD		LOD-1.9	1.7	LOD	
durum wheat	LOD-4.7	2.2	LOD		LOD	
spelt	2.3–3.1	2.3	LOD		LOD	
soft wheat	14.3–422.1	32.3	LOD-14.8	4.7	LOD	
tritordeum	40.3–738.0	164.2	24.6–15,389	262.3	LOD-245.5	LOD
barley	LOD-20.8	1.3	LOD-3.1	LOD	LOD-20.9	LOD
triticale	7.3–53.1	24.8	LOD-83.1	3.2	LOD	
rye	37.3–4,302	1345.1	33.8–711.8	75.4	LOD-741.8	47.6

Noteworthy, the overall contamination was particularly high in 2020 for all of the considered species, while lower EAs content was found in 2021 and 2022. Meteorological data recorded for the geographical area of cultivation clearly indicate a clear difference among the monthly rainfall (mm), the rainy days, and the growing degree days (GDD) in the three growing seasons, as reported in Table S1. In particular, while GDD values were similar, the rainfall recorded in April–June was 316 mm in 2020 versus 192 and 137 mm in 2021 and 2022, respectively. These data are consistent with the literature, indicating the relevant effect of frequent rainfall from the flowering to the ripening stage in the development of ergot sclerotia.^22^

**Figure 1:**
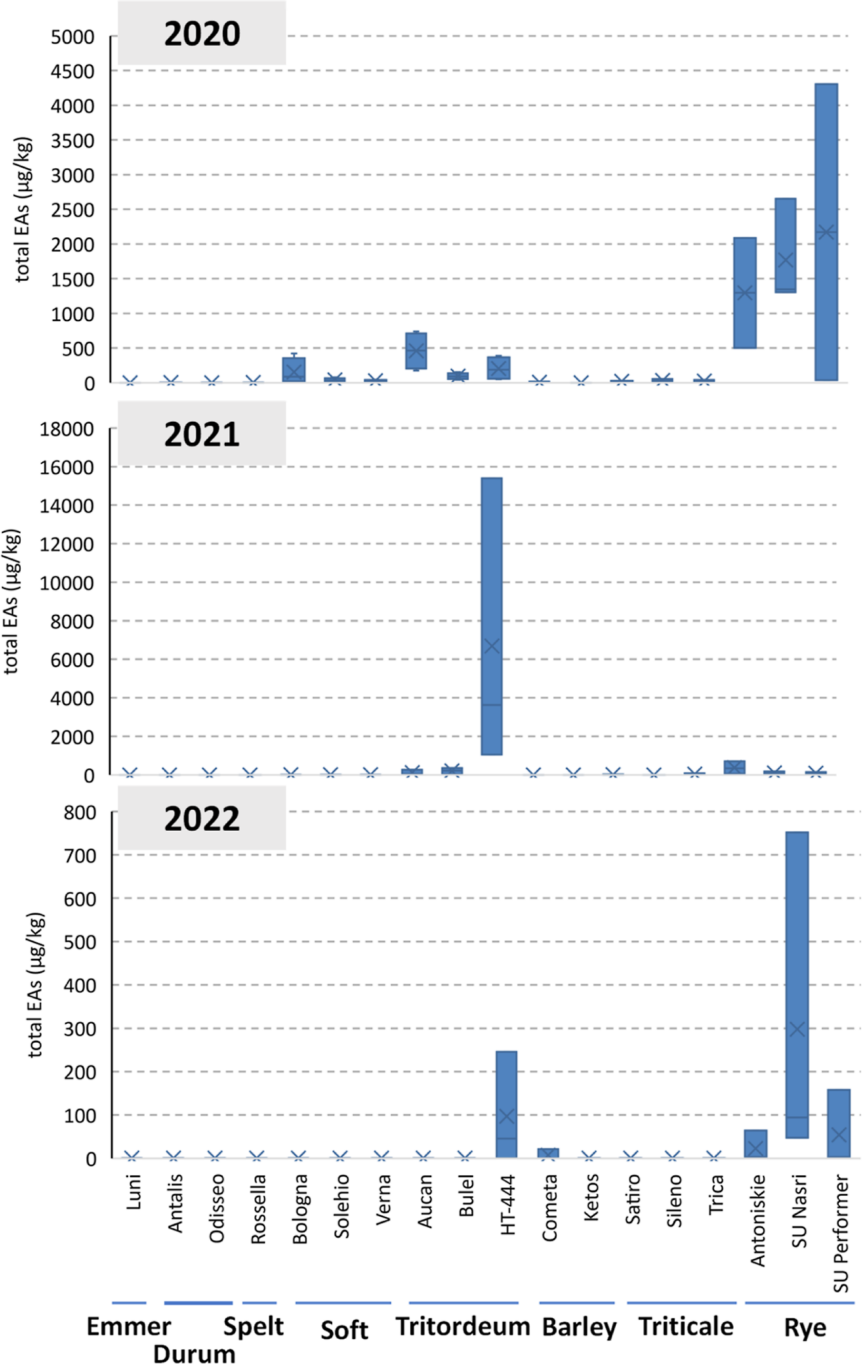
Box plot showing the total EA occurrence in the considered varieties over the 3 years of observation.

In general, 2020 was the year that showed a higher range of contamination for most cereals. On the contrary, in 2022 most of the samples were negative (below the LODs) with the exception of rye, tritordeum, and barley. In addition, the amount of contamination was notably higher for some crops such as rye and tritordeum reaching maximum concentrations of 4,302 and 15,389 μg/kg, respectively. Data of each individual variety are reported as a box plot in Figure 1.

A significantly higher EAs contamination was observed for the F_1_ hybrid cultivar of rye (Su Narsi and Su Performer, *p* < 0.0001) compared to the conventional varieties (Antoniskie), confirming data reported by Sardella et al., (2023)^29^ carried out in marginal growing areas. Mirdita and Miedaner (2009)^30^ highlighted that hybrid cultivars had a higher occurrence of poorly restored plants that shed less pollen, compared to conventional ones, which is instead characterized by full pollen shedding. *Claviceps* spp. showed a higher infection rate in florets that have not yet pollinated or just pollinated; thus, the hybrid, with a lower pollen production, was expected to be more prone to the disease than conventional varieties.^31^

At a species level, our study is consistent with the susceptibility ranking reported in the literature,^32,33^ with rye as the preferential host crop for *C. purpurea* and ergot sclerotia formation followed by soft wheat, durum wheat, and barley. Besides rye, in 2020 EAs were found in 100% of the soft wheat, triticale, and spelt samples, 75% of durum wheat, and 50% of barley samples. Although with a lower incidence, the same trend was observed in 2021, whereas the incidence was significantly lower in 2022.

Of particular interest are the high contamination results obtained for tritordeum, a hybrid crop obtained by crossing *T. turgidum* spp. *durum* and *H. chilense*. This crop was developed in 1977 by the Spanish National Research Council and was recently commercialized due to its interesting nutritional profile and higher resilience to hot and dry climates than wheat.^34,35^ Triticale, a hybrid crop obtained by crossing *Triticum* and *Secale* with the intent to combine the yield potential and quality of wheat with the environmental tolerance of rye, also presented a frequent incidence of contamination, in agreement with previous results.^32,33^ This may indicate for both hybrids a susceptibility tract inherited from the parent lines. When comparing soft and durum wheat over the three observation years, the latter showed lower incidence and significantly lower total EAs contamination (*p* = 0.0235).

Furthermore, our study highlighted a large interspecies variability, due to the inhomogeneous fungal spread that, in the case of ergot sclerotia, may lead to very high punctual concentration,^24^ with significant but still not conclusive differences in the comparison of crops over the three observation years. It is therefore difficult to draw any preliminary conclusion about cultivar-specific susceptibility starting from our data, which should be investigated on a fit-for-purpose trial.

### 3.2 Co-occurrence and Correlation of Individual Ergot Alkaloids

**Figure 2:**
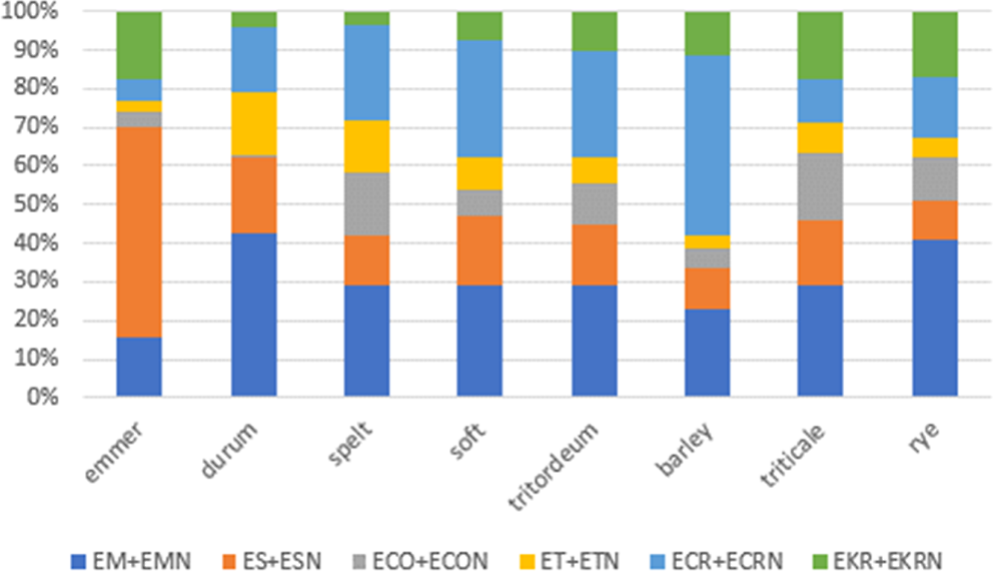
EA epimer pair distribution (%) within cereal crops.

To see whether different cereals showed a different distribution of EAs, the percentage ratio of each epimer pair was calculated over the overall sum of EAs. The distribution (%) is then reported in Figure 2. Such differences, although still preliminary, can be explained based on the potential modulation of EA biosynthesis exerted by the host crop. Interestingly, the largest difference in the EAs distribution was found in barley and emmer, showing premature and late flowering compared to other species, respectively (Table S5, Supporting Information). Therefore, such different distribution could also be ascribed to differences in the *Claviceps* populations, based on flowering and infection time, as already reported in the literature.^19^ In particular, barley is characterized by a large content of ECR and ECRN, while ES and ESN are the most abundant alkaloids in emmer.

**Figure 3:**
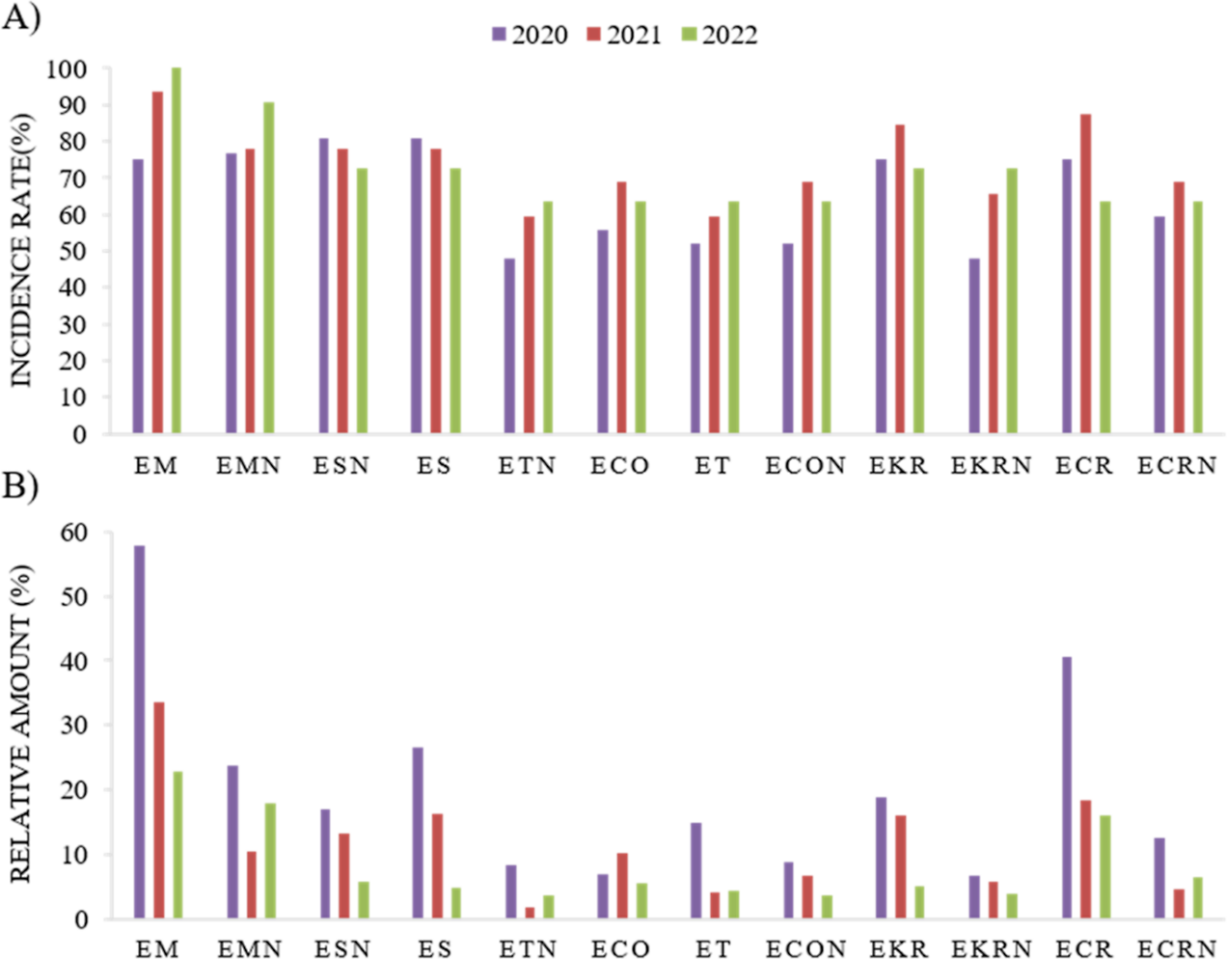
Evaluation of the positive samples over the three harvesting years: (A) incidence rate (%) of individual EAs, calculated as the percentage of samples containing a given EA; (B) relative amount (%) of the individual EAs, calculated as the percentage of a given EA over the total EAs.

On the other hand, the incidence rate (% occurrence of each EA over the total positive samples, Figure 3A) relative amount (% of each EA over the total EA content, Figure 3B) is reported in Figure 3. Interestingly, although the absolute contamination levels are highly different among years and species, the frequency of occurrence of single EAs is almost constant over time.

EM and EMN were the most frequently found compounds, especially in 2022, being present in more than 90% of the positive samples. These results are in line with other previous studies that also reported EM as the most common EA in cereal-based products from Italy.^18,19^ EKR, ECR, and ES appeared also as predominant EAs, also consistent with the literature.^2,3^ In general, the results demonstrated a large co-occurrence of EAs, with more than 50% of grain samples presenting all 12 EAs regardless of the harvest year and the species.

The distribution of the -ine and the -inine forms is highly correlated (*p* < 0.0001 for all the considered forms; data not shown) and stable over the years, with the only exception of EKR/EKRN, given that in 2022, with the lower EA occurrence, EKR incidence rate decreased, while the corresponding epimer form slightly increased (Figure 3A).

Regarding the relative amounts of the individual EAs, they were calculated as the ratio of the sum of the individual EA to the sum of the total EA concentration in the positive samples for each harvesting year and then divided by the number of positive samples in the corresponding year (Figure 3B). As expected, although the frequency of contamination of EKRN was similar or even slightly higher than EKR in 2022, the relative amount of the epimer was lower than the main EAs. In general, -inine forms are lower than -ine epimers in all the considered species, in agreement with the literature.^9,24^ The most prevalent form found in our samples was EM, followed by ES and ECR. This is partially in contrast with EFSA data, reporting ET as the prevalent EA in EU samples.^6,9^ This can be explained by taking into consideration the sensitivity issues often encountered for EM due to its poor peak shape. The EFSA data set presented indeed a high proportion of left-censored data (86%), with a cutoff value of 20 μg/kg. However, this issue was fixed in our study, allowing a LOQ of 0.2 μg/kg and, therefore, a more careful detection of all the EAs forms occurring in the considered sample set.

**Figure 4:**
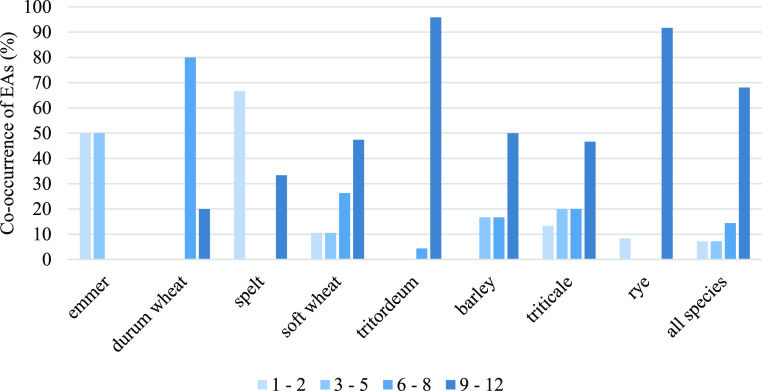
Co-occurrence of EAs. The number of EAs presented together in positive samples is considered as indicated in the legend.

The co-occurrence of EAs in positive samples (containing at least one EA > LOQ) of each cereal crop as well as in all positive samples together is shown in Figure 4. Of the 98 positive samples, only seven samples (7%) contained 1–2 EAs. The same percentage of samples contained a range of EAs between 3 and 5. 6–8 EAs were present in 14% of the positive samples. Surprisingly, a great percentage of the positive samples (68%) presented a higher number of EAs, above 9.

Although some differences in the co-occurrence of EAs were observed between species, in general, a higher percentage of positive samples presented a number of EAs between 9 and 12. Only in the cases of emmer and spelt did most of the positive samples present a lower number of EAs being below 5 EAs.

The current study reported the occurrence of EAs in winter cereals harvested over three years from an experimental field located in North Italy. This is the first open field study about ergot contamination in Italy and the first report on the occurrence of EAs in tritordeum and emmer. Results indicate a widespread occurrence of all the major EAs in all the considered cereal crops, especially under supportive meteorological conditions. Rye was confirmed to be the crop more susceptible to fungal infection, in particular, as far as the cultivation of hybrid variety is concerned. Overall, our data clearly indicate that the absence of ergot sclerotia does not imply that EA levels are within the current MLs, especially for soft wheat.

Collected data underlined the necessity to carry out further trials to identify agronomic practices and less susceptible varieties to decrease EAs occurrence in grains, especially in seasons with heavy rainfall conditions from flowering to the end of ripening.

## Supporting Information Available

This material is available of charge on the ACS Publications Web site. The Supporting Information is available free of charge at https://pubs.acs.org/doi/10.1021/acs.jafc.3c05612.

Cereal crop and variety information; LC–MS/MS analysis parameters; detailed information on ergot contamination; and flowering dates for each variety (PDF)

## References

[ref1] Krska R.; Crews C. Significance, Chemistry and Determination of Ergot Alkaloids: A Review. Food Addit. Contam.: Part A 2008, 25 (6), 722–731. 10.1080/02652030701765756. 18484300

[ref2] Agriopoulou S. Ergot Alkaloids Mycotoxins in Cereals and Cereal-Derived Food Products: Characteristics, Toxicity, Prevalence, and Control Strategies. Agronomy 2021, 11 (5), 931 10.3390/agronomy11050931.

[ref3] Malysheva S. V.; Larionova D. A.; Diana Di Mavungu J.; De Saeger S. Pattern and Distribution of Ergot Alkaloids in Cereals and Cereal Products from European Countries. World Mycotoxin J. 2014, 7 (2), 217–230. 10.3920/WMJ2013.1642.

[ref4] Paterson R. R. M.; Lima N. Further Mycotoxin Effects from Climate Change. Food Res. Int. 2011, 44 (9), 2555–2566. 10.1016/j.foodres.2011.05.038.

[ref5] Lombaert G. A. Liquid Chromatographic Method for the Determination of Ergot Alkaloids in Cereal Grains. Mycotoxin Protocols; Humana Press: NJ, 2000; Vol. 157, pp 215–224. 10.1385/1-59259-064-0:215 11051005

[ref6] Opinion of the Scientific Panel on Contaminants in the Food Chain [CONTAM] Related to Ergot as Undesirable Substance in Animal Feed. EFSA J., 2005, 3, 225. 10.2903/j.efsa.2005.225.

[ref7] Uhlig S.; Rangel-Huerta O. D.; Divon H. H.; Rolén E.; Pauchon K.; Sumarah M. W.; Vrålstad T.; Renaud J. B. Unraveling the Ergot Alkaloid and Indole Diterpenoid Metabolome in the Claviceps Purpurea Species Complex Using LC-HRMS/MS Diagnostic Fragmentation Filtering. J. Agric. Food Chem. 2021, 69 (25), 7137–7148. 10.1021/acs.jafc.1c01973. 34148344

[ref8] Coufal-Majewski S.; Stanford K.; McAllister T.; Blakley B.; McKinnon J.; Chaves A. V.; Wang Y. Impacts of Cereal Ergot in Food Animal Production. Front. Vet. Sci. 2016, 3, 15 10.3389/fvets.2016.00015. 26942186 PMC4766294

[ref9] Arcella D.; Gómez Ruiz J. Á.; Innocenti M. L.; Roldán R.; Human and Animal Dietary Exposure to Ergot Alkaloids. EFSA J. 2017, 15 (7), e04902 10.2903/j.efsa.2017.4902. 32625563 PMC7010019

[ref10] Hafner M.; Sulyok M.; Schuhmacher R.; Crews C.; Krska R. Stability and Epimerisation Behaviour of Ergot Alkaloids in Various Solvents. World Mycotoxin J. 2008, 1 (1), 67–78. 10.3920/WMJ2008.x008.

[ref11] Crews C. Analysis of Ergot Alkaloids. Toxins 2015, 7 (6), 2024–2050. 10.3390/toxins7062024. 26046699 PMC4488688

[ref12] Chung S. W. C. A Critical Review of Analytical Methods for Ergot Alkaloids in Cereals and Feed and in Particular Suitability of Method Performance for Regulatory Monitoring and Epimer-Specific Quantification. Food Addit. Contam.: Part A 2021, 38 (6), 997–1012. 10.1080/19440049.2021.1898679. 33784227

[ref13] Arroyo-Manzanares N.; Gámiz-Gracia L.; García-Campaña A. M.; Diana Di Mavungu J.; De Saeger S. Ergot Alkaloids: Chemistry, Biosynthesis, Bioactivity, and Methods of Analysis. In Fungal Metabolites; Mérillon J.-M., Ramawat K. G., Eds.; Reference Series in Phytochemistry; Springer International Publishing: Cham, 2017; pp 887–929.

[ref14] Commission Regulation (EU) 2023/915 of 25 April 2023 on Maximum Levels for Certain Contaminants in Food and Repealing Regulation (EC) No 1881/2006 (Text with EEA Relevance). 2023; Vol. 119. http://data.europa.eu/eli/reg/2023/915/oj/eng (accessed 2023-05-28).

[ref15] Schummer C.; Brune L.; Moris G. Development of a UHPLC-FLD Method for the Analysis of Ergot Alkaloids and Application to Different Types of Cereals from Luxembourg. Mycotoxin Res. 2018, 34 (4), 279–287. 10.1007/s12550-018-0322-5. 30117109

[ref16] Arroyo-Manzanares N.; De Ruyck K.; Uka V.; Gámiz-Gracia L.; García-Campaña A. M.; De Saeger S.; Diana Di Mavungu J. In-House Validation of a Rapid and Efficient Procedure for Simultaneous Determination of Ergot Alkaloids and Other Mycotoxins in Wheat and Maize. Anal. Bioanal. Chem. 2018, 410 (22), 5567–5581. 10.1007/s00216-018-1018-6. 29574560

[ref17] Veršilovskis A.; Mulder P. P. J.; Pereboom-de Fauw D. P. K. H.; de Stoppelaar J.; de Nijs M. Simultaneous Quantification of Ergot and Tropane Alkaloids in Bread in the Netherlands by LC-MS/MS. Food Addit. Contam., Part B 2020, 13 (3), 215–223. 10.1080/19393210.2020.1771777. 32482157

[ref18] Lattanzio V. M. T.; Verdini E.; Sdogati S.; Caporali A.; Ciasca B.; Pecorelli I. Undertaking a New Regulatory Challenge: Monitoring of Ergot Alkaloids in Italian Food Commodities. Toxins 2021, 13 (12), 871 10.3390/toxins13120871. 34941709 PMC8708126

[ref19] Debegnach F.; Patriarca S.; Brera C.; Gregori E.; Sonego E.; Moracci G.; De Santis B. Ergot Alkaloids in Wheat and Rye Derived Products in Italy. Foods 2019, 8 (5), 150 10.3390/foods8050150. 31052444 PMC6560453

[ref20] Kodisch A.; Oberforster M.; Raditschnig A.; Rodemann B.; Tratwal A.; Danielewicz J.; Korbas M.; Schmiedchen B.; Eifler J.; Gordillo A.; et al. Covariation of Ergot Severity and Alkaloid Content Measured by HPLC and One ELISA Method in Inoculated Winter Rye across Three Isolates and Three European Countries. Toxins 2020, 12 (11), 676 10.3390/toxins12110676. 33114663 PMC7692364

[ref21] Huybrechts B.; Malysheva S. V.; Masquelier J. A Targeted UHPLC-MS/MS Method Validated for the Quantification of Ergot Alkaloids in Cereal-Based Baby Food from the Belgian Market. Toxins 2021, 13 (8), 531 10.3390/toxins13080531. 34437402 PMC8402575

[ref22] Miller J. D. Changing Patterns of Fungal Toxins in Crops: Challenges for Analysts. J. AOAC Int. 2016, 99 (4), 837–841. 10.5740/jaoacint.16-0110. 27455926

[ref23] Uhlig S.; Eriksen G. S.; Hofgaard I. S.; Krska R.; Beltrán E.; Sulyok M. Faces of a Changing Climate: Semi-Quantitative Multi-Mycotoxin Analysis of Grain Grown in Exceptional Climatic Conditions in Norway. Toxins 2013, 5 (10), 1682–1697. 10.3390/toxins5101682. 24084167 PMC3813906

[ref24] Orlando B.; Maumené C.; Piraux F. Ergot and Ergot Alkaloids in French Cereals: Occurrence, Pattern and Agronomic Practices for Managing the Risk. World Mycotoxin J. 2017, 10 (4), 327–338. 10.3920/WMJ2017.2183.

[ref25] Coufal-Majewski S.; Stanford K.; McAllister T.; Wang Y.; Blakley B.; McKinnon J.; Chaves A. V. Effects of Pelleting Diets Containing Cereal Ergot Alkaloids on Nutrient Digestibility, Growth Performance and Carcass Traits of Lambs. Anim. Feed Sci. Technol. 2017, 230, 103–113. 10.1016/j.anifeedsci.2017.06.006. PMC5744125 29257065

[ref26] Coufal-Majewski S.; Stanford K.; McAllister T.; Wang Y.; Blakley B.; McKinnon J.; Swift M. L.; Chaves A. V. Effects of Continuously Feeding Diets Containing Cereal Ergot Alkaloids on Nutrient Digestibility, Alkaloid Recovery in Feces, and Performance Traits of Ram Lambs. Toxins 2017, 9 (12), 405 10.3390/toxins9120405. 29257065 PMC5744125

[ref27] Ávila C. M.; Rodríguez-Suárez C.; Atienza S. G. Tritordeum: Creating a New Crop Species-The Successful Use of Plant Genetic Resources. Plants 2021, 10 (5), 1029 10.3390/plants10051029. 34065483 PMC8161160

[ref28] Carbonell-Rozas L.; Gámiz-Gracia L.; Lara F. J.; García-Campaña A. M. Determination of the Main Ergot Alkaloids and Their Epimers in Oat-Based Functional Foods by Ultra-High Performance Liquid Chromatography Tandem Mass Spectrometry. Molecules 2021, 26 (12), 3717 10.3390/molecules26123717. 34207051 PMC8234484

[ref29] Sardella C.; Capo L.; Adamo M.; Donna M.; Ravetto Enri S.; Vanara F.; Lonati M.; Mucciarelli M.; Blandino M. The Cultivation of Rye in Marginal Alpine Environments: A Comparison of the Agronomic, Technological, Health and Sanitary Traits of Local Landraces and Commercial Cultivars. Front. Plant Sci. 2023, 14, 1130543 10.3389/fpls.2023.1130543. 37235035 PMC10208067

[ref30] Mirdita V.; Miedaner T. Resistance to Ergot in Self-Incompatible Germplasm Resources of Winter Rye. J. Phytopathol. 2009, 157 (6), 350–355. 10.1111/j.1439-0434.2008.01499.x.

[ref31] Miedaner T.; Geiger H. Biology, Genetics, and Management of Ergot (Claviceps Spp.) in Rye, Sorghum, and Pearl Millet. Toxins 2015, 7 (3), 659–678. 10.3390/toxins7030659. 25723323 PMC4379517

[ref32] Babič J.; Tavčar-Kalcher G.; Celar F. A.; Kos K.; Červek M.; Jakovac-Strajn B. Ergot and Ergot Alkaloids in Cereal Grains Intended for Animal Feeding Collected in Slovenia: Occurrence, Pattern and Correlations. Toxins 2020, 12 (11), 730 10.3390/toxins12110730. 33233446 PMC7700445

[ref33] Schwake-Anduschus C.; Lorenz N.; Lahrssen-Wiederholt M.; Lauche A.; Dänicke S. German Monitoring 2012–2014: Ergot of Claviceps Purpurea and Ergot Alkaloids (EA) in Feedingstuffs and Their Toxicological Relevance for Animal Feeding. J. Verbraucherschutz Lebensmittelsicherh. 2020, 15 (4), 321–329. 10.1007/s00003-020-01298-7.

[ref34] Vaquero L.; Comino I.; Vivas S.; Rodríguez-Martín L.; Giménez M. J.; Pastor J.; Sousa C.; Barro F. Tritordeum: A Novel Cereal for Food Processing with Good Acceptability and Significant Reduction in Gluten Immunogenic Peptides in Comparison with Wheat. J. Sci. Food Agric. 2018, 98 (6), 2201–2209. 10.1002/jsfa.8705. 28963718

[ref35] Martín A.; Alvarez J. B.; Martín L.; Barro F.; Ballesteros J. The Development of Tritordeum: A Novel Cereal for Food Processing. J. Cereal. Sci. 1999, 30 (2), 85–95. 10.1006/jcrs.1998.0235.

