# Supplementary material for: Occurrence of Ergot Alkaloids in Major and Minor Cereals from Northern Italy: A Three Harvesting Years Scenario
Source: J Agric Food Chem. 2023 Oct 16;71(42):15821–8. doi: 10.1021/acs.jafc.3c05612 (PMC10603808; doi:10.1021/acs.jafc.3c05612)
Supplement: Supplementary file 1 — jf3c05612_si_001.pdf [file jf3c05612_si_001.pdf]

SUPPORTING INFORMATION

Occurrence of ergot alkaloids in major and minor cereals from northern Italy: a three harvesting years scenario

Laura Carbonell-Rozas[1#], Arianna Alabrese[1], Raffaele Meloni[2,] Laura Righetti[1,3,4*], Massimo Blandino[2*], Chiara Dall'Asta[1]

[1]Department of Food and Drug, University of Parma, Viale delle Scienze 27/A, 43124 Parma (Italy)
[2] Department of Agricultural, Forest and Food Sciences, University of Turin, Largo Paolo Braccini 2, 10095 Grugliasco (Italy).
[3] Laboratory of Organic Chemistry, Wageningen University, Wageningen 6708 WE, the Netherlands;
[4] Wageningen Food Safety Research, Wageningen University & Research, Wageningen 6700 AE, the Netherlands

#Present address: Department of Chemistry and Physics, Research Centre for Mediterranean Intensive Agrosystems and Agrifood Biotechnology (CIAIMBITAL), Agrifood Campus of International Excellence (ceiA3), University of Almeria, E-04120 Almeria, Spain

*Corresponding authors:

Laura Righetti: laura.righetti@wur.nl
Massimo Blandino: massimo.blandino@unito.it

Table S1. List of cereal crops and varieties considered in this study.

Crop	Specie	Ploidy level[1]	Variety	Specific trait	Seed company	Year of release
emmer	*Triticum turgidum* spp. *dicoccum*	tetraploid (AABB)	Luni		S.I.S., San Lazzaro di Savena, Italy	2002
durum wheat	*Triticum turgidum spp. durum*	tetraploid (AABB)	Odisseo		Produttori Sementi Bologna, Argelato, Italy	2011
		tetraploid (AABB)	Antalis		CGS Sementi S.p.A., Acquasparta, Italy	2014
spelt	*Triticum aestivum* spp. *spelta*	hexaploid (AABBDD)	Rossella		Apsovsementi, Voghera, Italy	2016
soft wheat	*Triticum aestivum* spp. *aestivum*	hexaploid (AABBDD)	Verna		Italian local landrace	1953
			Bologna		S.I.S., San Lazzaro di Savena, Italy	2002
			Solehio		Agroalimentare Sud Spa, Melfi, Italy	2008
tritordeum	×*Tritordeum martinii*	hexaploid (AABBHchHch)	Aucan		Vivagrain, Barcellona, Spain	2013
			Bulel		Vivagrain, Barcellona, Spain	2015
			Coique		Vivagrain, Barcellona, Spain	-
barley	*Hordeum vulgare*	diploid (HH)	Ketos	six-row cv	Limagrain Italia Spa, Fidenza, Italy	2002
			Cometa	two-row cv	Apsovsementi, Voghera, Italy	2006
triticale	×*Triticosecale*	hexaploid (AABBRR)	Trica		S.I.S., San Lazzaro di Savena, Italy	1999
			Sileno		Apsovsementi, Voghera, Italy	2016
			Satiro		Apsovsementi, Voghera, Italy	2017
rye	*Secale cereale*	diploid (RR)	Antoninskie		Poznańska Hodowla Roślin Sp. z.o.o., Tulce, Poland	2014
			Su Nasri	F1 hybrid	Saaten-Union GmvH, Isernhagen, Germany	2015
			Su Performer	F1 hybrid	Saaten-Union GmvH, Isernhagen, Germany	2016

[1] Ploidy level = number of sets of chromosomes.

Table S2. Monthly rainfall, rainy days, and growing degree-days (GDD) from the sowing (November) to the end of ripening stage (June) in the 3 growing seasons.

Growing season		rainfall (mm)	rainy days (d)	GDD[1] (Σ °Cd^{-1})
2019 – 2020	Nov	314	12	249
	Dec	132	8	193
	Jan	5	0	168
	Feb	1	0	229
	Mar	62	4	285
	Apr	81	7	414
	May	122	9	579
	June	113	7	624
April - June		*316*	*23*	*1617*
2020 – 2021	Nov	4	0	277
	Dec	79	8	144
	Jan	116	8	128
	Feb	29	3	203
	Mar	8	1	286
	Apr	37	7	353
	May	69	6	501
	June	86	7	674
April - June		*192*	*20*	*1528*
2021 – 2022	Nov	134	10	244
	Dec	17	2	149
	Jan	6	1	152
	Feb	9	1	196
	Mar	13	2	265
	Apr	34	3	384
	May	82	6	621
	June	21	3	694
April - June		*137*	*12*	*1699*

Source: Rete Agrometeorologica del Piemonte - Regione Piemonte - Assessorato Agricoltura - Settore Fitosanitario, sezione di Agrometeorologia. [1]Accumulated growing degree days for each experiment using a 0°C base value.

Table S3. LC-MS/MS parameters for the target compounds.

EA	Retention time (min)	Precursor ion (*m/z*) [M+H] +	Product Ions (*m/z*)	Collision Energy (eV)
**EM**	0.74	326	208	30
			223	25
**EMN**	0.97	326	208	28
			223	26
**ESN**	4.03	548	223	32
			530	15
**ES**	4.32	548	208	42
			223	34
**ETN**	5.00	582	208	46
			268	25
**ECO**	4.96	562	277	27
			544	14
**ET**	5.13	582	208	47
			223	36
**ECON**	6.14	562	305	28
			558	14
**EKR**	6.14	576	223	33
			564	14
**ECR**	6.31	610	208	48
			223	48
**EKRN**	6.78	576	208	43
			223	33
**ECRN**	6.98	610	305	29
			592	13

EM: ergometrine, EMN: ergometrinine, ES: ergosine, ESN: ergosinine, ET: ergotamine, ETN: ergotaminine, ECO: ergocornine, ECON: ergocorninine, EKR: ergocryptine, EKRN: ergocryptinine, ECR: ergocristine, ECRN: ergocristinine

Table S4. EAs contamination in cereal samples across 2020-2021-2022 seasons for the different cereal varieties.

Species	Variety	Concentration 2020 (µg/Kg)		Concentration 2021 (µg/Kg)		Concentration 2022 (µg/Kg)	
		Mean	Range	Mean	Range	Mean	Range
Rye	Antoninskie	107.87	5.05-694.33	30.48	2.47-296.65	8.45	0.61-18.24
	Su Nasri	147.26	6.72-892.61	8.36	2.28-57.85	45.85	1.99-366.74
	Su Performer	180.82	2.35-1885.10	6.93	2.17-63.01	18.93	0.79-45.07
Tritordeum	Aucan	43.69	3.51-404.75	9.44	1.80-80.44	0.00	0.00
	Bulel	8.48	2.50-56.28	17.76	2.05-148.67	0.00	0.00
	HT-444	17.76	2.17-95.90	557.29	13.80-4634.06	21.53	1.56-109.83
Common wheat	Bologna	14.52	1.33-134.69	2.77	2.06-2.64	0.00	0.00
	Solehio	3.68	1.56-26.20	2.44	2.08-2.99	0.00	0.00
	Verna	3.25	1.12-9.93	3.13	2.06-2.25	0.00	0.00
Triticale	Sileno	3.34	1.62-10.07	0.00	0.00	0.00	0.00
	Trica	3.82	1.90-11.78	1.95	0.18-34.01	0.00	0.00
	Satiro	2.52	1.80-3.25	4.87	0.06-3.71	0.00	0.00
Dicoccum	Luni	0.00	0.00-0.00	1.15	1.58-2.41	0.00	0.00
Spelt	Rossella	2.55	1.26-7.46	0.00	0.00	0.00	0.00
Durum wheat	Antalis	3.17	0.36-16.75	0.00	0.00	0.00	0.00
	Odisseo	3.19	1.65-5.62	0.00	0.00	0.00	0.00
Barley	Cometa	9.73	0.28-121.92	3.05	0.88-6.36	3.80	1.40-5.40
	Ketos	0.00	0.00-0.00	0.00	0.00	0.00	0.00

Table S5. Date of flowering (growth stage 61 according to the BBCH scale) of the compared varieties and crops in the considered harvested years.

**Crop**	**Variety**	**2020**	**2021**	**2022**
emmer	Luni	12 May 2020	16 May 2021	15 May 2022
durum wheat	Odisseo	3 May 2020	8 May 2021	8 May 2022
	Antalis	5 May 2020	12 May 2021	11 May 2022
spelt	Rossella	2 May 2020	3 May 2021	4 May 2022
soft wheat	Verna	11 May 2020	14 May 2021	13 May 2022
	Bologna	3 May 2020	6 May 2021	7 May 2022
	Solehio	5 May 2020	8 May 2021	9 May 2022
tritordeum	Aucan	3 May 2020	7 May 2021	8 May 2022
	Bulel	6 May 2020	11 May 2021	11 May 2022
	Coique	5 May 2020	12 May 2021	11 May 2022
barley	Ketos	29 April 2020	28 April 2021	1 May 2022
	Cometa	23 April 2020	21 April 2021	26 April 2022
triticale	Trica	29 April 2020	27 April 2021	1 May 2022
	Sileno	6 May 2020	6 May 2021	8 May 2022
	Satiro	3 May 2020	5 May 2021	7 May 2022
rye	Antoninskie	6 May 2020	9 May 2021	10 May 2022
	Su Nasri	6 May 2020	11 May 2021	11 May 2022
	Su Performer	4 May 2020	10 May 2021	10 May 2022

Field experiments carried out in North Italy; date of sowing 6 November 2019, 3 November 2020 and 9 November 2021; date of harvest 29 June 2020, 5 July 2021.
